# Machine learning-based prediction of intraoperative hypoxemia for pediatric patients

**DOI:** 10.1371/journal.pone.0282303

**Published:** 2023-03-01

**Authors:** Jung-Bin Park, Ho-Jong Lee, Hyun-Lim Yang, Eun-Hee Kim, Hyung-Chul Lee, Chul-Woo Jung, Hee-Soo Kim

**Affiliations:** 1 Department of Anesthesiology and Pain Medicine, Seoul National University College of Medicine, Seoul, Republic of Korea; 2 Department of Anesthesiology and Pain Medicine, Seoul National University Hospital, Seoul, Republic of Korea; Hanyang University, REPUBLIC OF KOREA

## Abstract

**Background:**

Reducing the duration of intraoperative hypoxemia in pediatric patients by means of rapid detection and early intervention is considered crucial by clinicians. We aimed to develop and validate a machine learning model that can predict intraoperative hypoxemia events 1 min ahead in children undergoing general anesthesia.

**Methods:**

This retrospective study used prospectively collected intraoperative vital signs and parameters from the anesthesia ventilator machine extracted every 2 s in pediatric patients undergoing surgery under general anesthesia between January 2019 and October 2020 in a tertiary academic hospital. Intraoperative hypoxemia was defined as oxygen saturation <95% at any point during surgery. Three common machine learning techniques were employed to develop models using the training dataset: gradient-boosting machine (GBM), long short-term memory (LSTM), and transformer. The performances of the models were compared using the area under the receiver operating characteristics curve using randomly assigned internal testing dataset. We also validated the developed models using temporal holdout dataset. Pediatric patient surgery cases between November 2020 and January 2021 were used. The performances of the models were compared using the area under the receiver operating characteristic curve (AUROC).

**Results:**

In total, 1,540 (11.73%) patients with intraoperative hypoxemia out of 13,130 patients’ records with 2,367 episodes were included for developing the model dataset. After model development, 200 (13.25%) of the 1,510 patients’ records with 289 episodes were used for holdout validation. Among the models developed, the GBM had the highest AUROC of 0.904 (95% confidence interval [CI] 0.902 to 0.906), which was significantly higher than that of the LSTM (0.843, 95% CI 0.840 to 0.846 *P* < .001) and the transformer model (0.885, 95% CI, 0.882–0.887, *P* < .001). In holdout validation, GBM also demonstrated best performance with an AUROC of 0.939 (95% CI 0.936 to 0.941) which was better than LSTM (0.904, 95% CI 0.900 to 0.907, *P* < .001) and the transformer model (0.929, 95% CI 0.926 to 0.932, *P* < .001).

**Conclusions:**

Machine learning models can be used to predict upcoming intraoperative hypoxemia in real-time based on the biosignals acquired by patient monitors, which can be useful for clinicians for prediction and proactive treatment of hypoxemia in an intraoperative setting.

## Introduction

Intraoperative hypoxemia is an urgent clinical situation warranting immediate intervention to prevent permanent complications such as hypoxic brain injury [1]. Hypoxemia is more prevalent in pediatric patients owing to their significant physiologic limitations concerning apnea tolerance [2]. Children have a smaller functional capacity and increased metabolic requirements and oxygen consumption, resulting in faster progression of hypoxemia and slower recovery [1, 2]. In previous studies, the reported prevalence of intraoperative hypoxemia (oxygen saturation [SpO_2_] <95%) during general anesthesia in children aged <16 years was 11.1–22%, and the reported incidences were considerably higher in the younger age group [2–6].

Although the incidence of mortality associated with anesthesia-related cardiac arrest in children has been reduced over the past few decades, 27% of perioperative arrests are attributed to respiratory causes according to the Pediatric Perioperative Cardiac Arrest registry [7–9]. Anesthesia-related risk factors and several clinical features, such as age ≤3 years, higher American Society of Anesthesiologists physical status, morbid obesity, and preexisting pulmonary disorder are validated for intraoperative hypoxemia prediction [4]. However, predicting the occurrence of hypoxemic events in real time during general anesthesia, even in children without any known risk factors, is still challenging [3].

Recent advances in machine learning techniques enable the construction of accurate prediction models for various medical applications [10–14]. Such techniques identify hidden patterns in large datasets and analyze correlations among variables or features. In previous studies, machine learning techniques have been used to predict clinical outcomes, such as sepsis, delirium, acute kidney injury, and intraoperative hypotension using arterial pressure waveforms [13, 15–17]. However, to the best of our knowledge, no study has investigated the use of machine learning algorithms for predicting intraoperative hypoxemia in children.

In this study, we hypothesized that machine learning model and complex feature extraction techniques could be utilized to predict intraoperative hypoxemia events 1 minute ahead in children undergoing surgery under general anesthesia. Unlike previous machine learning model approaches integrating large datasets into a single risk, continuous performance using not only static data, such as patient and procedure features, but also including high-fidelity real-time data is important for determining targeted interventions in a clinical setting. Thus, we aimed to develop a machine learning model that predicts hypoxia 1 min ahead during general anesthesia in pediatric patients and verify the performance of our model using a pediatric vital signs registry dataset.

## Materials and methods

### Data source and ethics

This retrospective study was approved by the Institutional Review Board (IRB) of Seoul National University Hospital, Seoul, Korea, (number 2011-208-1179; Chairperson, Byung-joo Park; Date of approval, 15 December 2020); the requirement for written informed patient consent was waived by the IRB owing to the retrospective nature of the study.

### Data collection

A retrospective analysis of prospectively collected intraoperative vital sign registry (VitalDB) data was performed. A total of 13,130 patients aged ≤18 years, including newborns and premature infants, who underwent surgery under general anesthesia between January 2019 and October 2020 at a tertiary referral center, Seoul National University Children’s Hospital, Seoul, South Korea, were included in this study. The exclusion criteria were 1) aged ≥19 years, 2) surgery performed with only regional or local anesthesia, peripheral nerve block, or monitored anesthesia care, 3) patients undergoing cardiac surgery using cardiopulmonary bypass, 3) those who needed one-lung ventilation for surgery, 4) inevitable apneic time during surgical procedure, such as airway surgery including tracheal resection, and 5) preoperative SpO_2_ of < 95%.

Demographic data such as age, sex, height, and weight were collected from the patients’ electronic medical records. The intraoperative vital signs data used in this study were collected using various medical devices during general anesthesia using the Vital Recorder Programme (available at https://vitaldb.net; accessed March 4, 2021). Data on noninvasive blood pressure or arterial pressure (if possible), electrocardiography, photoplethysmography, capnography waveform, and parameters from the anesthesia ventilator machine were used to identify genuine hypoxemic events. Changes in ventilator parameters included increases in peak inspiratory pressure (PIP), decreases in tidal volume (TV), abrupt changes of end tidal carbon dioxide concentration (EtCO_2_) and changes of fraction of inspired oxygen (FiO_2_). The ventilator machine parameters included The SpO_2_, EtCO_2_, FiO_2_, TV, and PIP measurement were extracted every 2 s as an instantaneous value.

### Data preparation

Hypoxemia was defined as SpO_2_ less than 95% during general anesthesia regardless of the duration. The limit of 95% was selected in line with the definition of hypoxemia of perioperative respiratory adverse events [5]. All 13,130 vitalDB records were manually checked for the detection of hypoxic episodes by an anesthesiologist. The data retrieved from the VitalDB database were rechecked manually by the second anesthesiologist for quality control. The observed events were marked and annotated in all the databases. The true hypoxemia was verified by SpO_2_ value, electronic medical records, vital signs, including heart rate, blood pressure, arterial blood pressure waveform, and pulse oximetry, plethysmographic waveform, and ventilator parameters, including EtCO_2_ curve, PIP, and TV. Genuine hypoxemic events were discriminated using the following exclusion criteria: 1) when the measured pulse oximetry pulse rate differed by more than 20% from the electrocardiogram heart rate, the associated SpO_2_, [2] 2) when anesthesiologists in charge recorded inaccurate measurement of oxygen saturation, and 3) when plethysmogram waveforms were severely distorted owing to position change or external pressure so that the signal quality reported in the monitor was severely low [2]. Artifacts that were mostly caused by motion, positioning, electrocautery, sensor dislodgement, and low peripheral perfusion were not annotated as hypoxemia and were retained in the dataset without further processing. Periods of hypoxemic episodes, divided into induction, maintenance, and emergence, were annotated in each database to determine the incidence of intraoperative hypoxemia in each period. We also investigated the total time of hypoxemia, duration from the minimum saturation to recovery until the saturation recovered to the initial value where saturation began to gradually decrease, and the cause of the hypoxemia episode suggested by the anesthesiologists based on various indicators. Patients were assigned to one of the four groups to confirm the incidence of the hypoxemic episodes stratified by age: 0–28 days (neonate), 29 days to 12 months (infant), 1–7 years, and 8–18 years [2]. Demographic data including age, sex, height, and weight were analyzed in each group.

Several parameters, such as SpO_2_, EtCO2, FiO_2_, TV, and PIP included in 1-minute length of segments from the determined hypoxemia episodes to the corresponding 1 minute ahead, were extracted. If a hypoxemic event defined as SpO_2_ dropping below 95%, in the upcoming 1 min was observed, the target was assigned as 1; it was assigned 0 otherwise. Note that target 1 was assigned for the segment where the input SpO_2_ was less than 95% while being included in the hypoxemia episode. Moreover, the patient’ demographic information (age, sex, height, and weight) was extracted corresponding to the segment and the target.

### Model training

Hypoxemia predictions were made for a window of 1 minute into the future. A positive label was considered if SpO_2_ was <95% at any point, otherwise it was considered negative. The machine learning algorithm was trained using these training labels at all time points in time.

The patients were randomly categorized into the training dataset (80%) and testing dataset (20%). Note that each dataset had a completely disjointed set of patients. For model training, patient demographics (age, sex, height, and weight) and 2 second interval data (SpO_2_, EtCO2, FiO_2_, TV, and PIP) were used as the input segment and the upcoming hypoxemia event was used as the output.

We considered three machine learning or deep learning algorithms: Gradient-boosting machine (GBM), long short-term memory (LSTM), and transformer. Each algorithm was searched with candidates of its own hyperparameters. The hyperparameters for the three machine learning or deep learning model were as follows. GBM, the most representative tree-based ensemble machine learning technique, was evaluated using the hyperparameters of {number of trees [2000], max depth: [3,4,5], subsampling rate: [0.5, 0.8]}. LSTM was searched using the hyperparameters of {number of hidden layers: [1,2], number of hidden nodes: [16, 32, 64], number of dense nodes: [16, 32, 64, 128], dropout rate: [0.2, 0.5]}. We assessed the transformer architecture using the hyperparameters of {number of filters: [16, 32, 64], number of heads: [2,3,4], embedded dimension: [16, 32, 64], number of convolutional layers: [1,2,3,4], number of transformer layers: [1,2,3], dropout rate: [0.1, 0.2]}.

GBM performs tree-based residual fitting and is known to function well even with many types of variables. Owing to its intrinsic limitations of not being able to consider time-series data, only the first event of each case was used for training and testing. We considered the LSTM-based deep learning model to exploit the full time-series data. This model is composed of recurrent paths and memory cells, such that the feature information of past data can be preserved to the same extent as the input length considered. LSTM was trained with intact input variables consisting of patient demographics and 2-second interval data. Lastly, the transformer-based deep learning model was developed, which is being touted as a good deep learning architecture for various data types [18–22]. Transformer-based deep learning model was trained using whole input variables as in the LSTM model training.

The input segment corresponding to a hypoxemia event is extremely rare; therefore, the collected data could be imbalanced. Since the machine learning models are only as good as the data quality that it is based on, it is necessary to handle the imbalance of the collected data. Machine learning models perform optimization on a given loss function for training. In the presence of imbalanced data such as the ratio of positive and negative of 1:99, the model can obtain 99% accuracy by merely negative prediction with a sufficiently good loss value. Since this is not desired, we added class weight to scale the importance of loss function of the positive label. Since the ratio of the hypoxemia and non-hypoxemia event differed by approximately 100 times in our dataset, we added class weight of 100 for the hypoxemia event.

Five-fold cross validation was performed to identify the optimal hyperparameter and the optimal combination of input variables. The training set was divided into five subsets and the model was trained sequentially using the remaining subsets with the exception of one, and model performance was evaluated using the mean area under the receiver operating characteristic curve (AUROC) using the excluded subset. Finally, the hyperparameter and variable combination with the largest mean AUROC was selected.

### Model holdout validation

**Fig 1:**
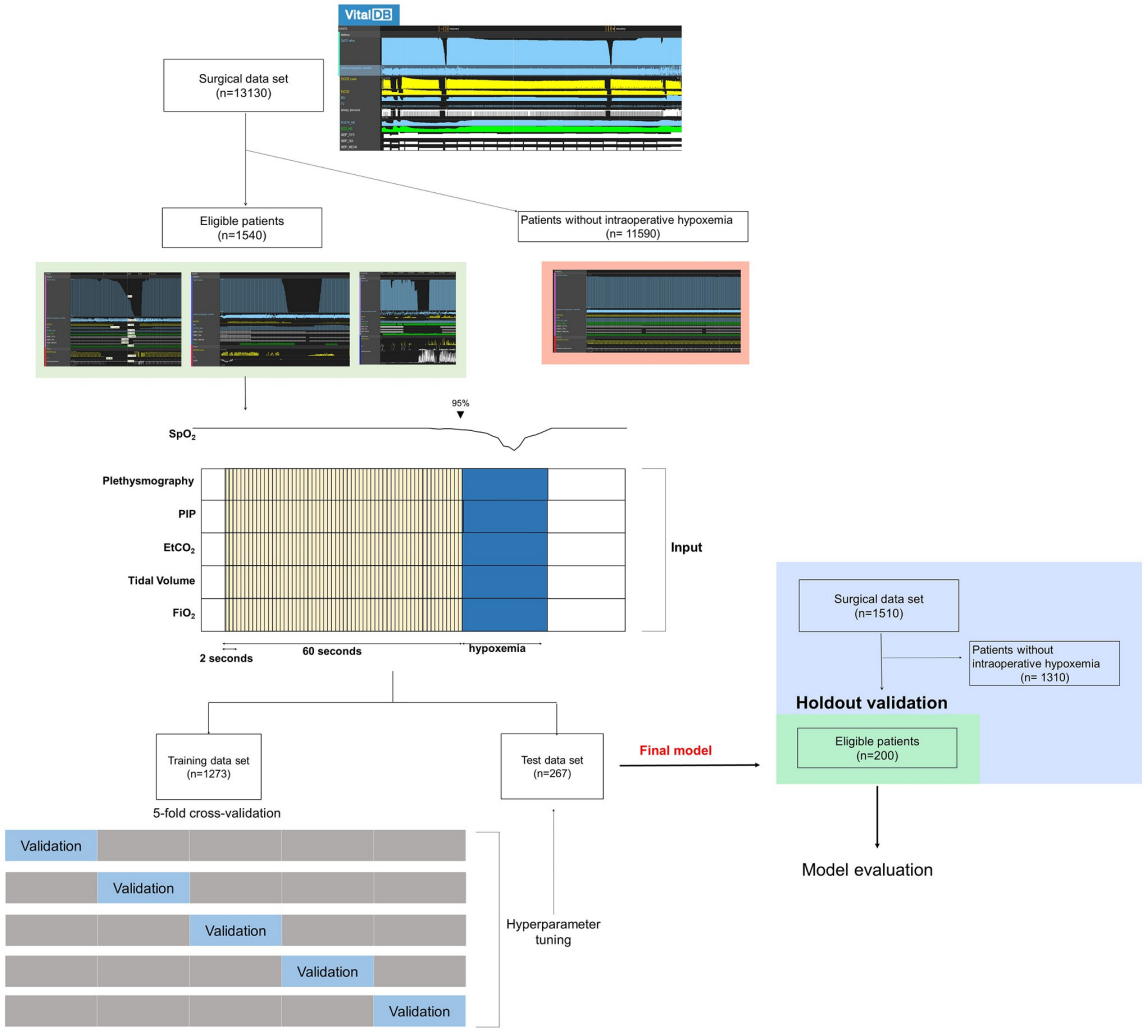
Flow chart presenting patient selection and data analysis. PIP, Peak Inspiratory Pressure; EtCO_2_ End tidal CO_2_; FiO_2_, Fraction of inspired oxygen.

Model holdout validation was performed on the holdout dataset that was not used for training or testing. Note that the final model was produced using training, validation, testing dataset from eligible 1540 patients, and additional holdout validation were performed with finalized model to prove our model’s reliability (Fig 1). A total of 1,510 vitalDB datasets between November 2020 and January 2021 from Seoul National University Children’s Hospital were used to evaluate the predictive power of our models on unseen separate holdout and temporal validation sets. Finally, 200 eligible patients were used for holdout validation. The data for holdout validation were extracted using the same rule for training dataset (see *Data preparation* section), except the filtering rule for the segment already having hypoxemia. The input segments that have SpO_2_ lower than 95% were filtered out to exclude cases that are easy to predict because patients who already have hypoxemia are more likely to have hypoxemia after 1 minute. The performances of each of the final models were assessed in the validation cohort.

Model training, testing, and holdout validation were performed using Python 3.8.0 programming language with XGBoost v1.5.0 and TensorFlow v2.7.0.

### Statistical analysis and model development

Normality was assessed using the Shapiro–Wilk W-test. Data are expressed as the mean and standard deviation for parametric variables and as frequencies/percentages for nonparametric variables. Comparisons of baseline demographics were performed using Pearson’s chi-square test for categorical variables and the Mann–Whitney U-test for continuous variables.

A receiver-operative characteristic curve analysis with 95% confidence intervals (CIs) and precision-recall curves was performed to assess model performance. The AUROC and area under precision-recall curve (AUPRC) were calculated for comparison. The performance of the AUROC was considered high (AUROC > 0.9), moderate (AUROC = 0.7–0.9), or low (AUROC = 0.5–0.7) [23]. The testing area under the curve (AUC) values corresponding to the different models were compared using a paired Delong test [15]. Moreover, sensitivity and specificity were evaluated for each model. The sensitivity of the model refers to the ability of the model to correctly identify those patients within hypoxemia:

$$Sensitivity=\frac{True\;positive}{True\;positive+False\;negative}$$

The specificity of the model refers to the ability of the test to correctly identify those patients without hypoxemia:

$$Specificity=\frac{True\;negative}{False\;positive+True\;negative}”$$

A calibration plot was visualized to evaluate the agreement of the predictions and observations within a range of percentiles of the predicted values using the three models [24].

Statistical analyses were performed using the Python 3.8 programming language and R software (version 3.6.3; R Foundation for Statistical Computing). *P*<0.05 was considered statistically significant.

## Results

### Baseline characteristics

Of the 13,130 patient records identified, the records of 1,540 (11.73%) patients with 2,367 episodes of intraoperative hypoxemia were included in the model development (Fig 1). The baseline patient characteristics are shown in Table 1. The patient demographics, including age, sex, height, and weight, were comparable between the training, test, and holdout validation datasets.

**Table 1:** Baseline characteristics in the dataset.

		Total (n = 1540)	Training set (n = 1273)	Testing set (n = 267)	Validation set (n = 200)	*p*-Value
**Age**		5.30 ± 4.73	5.32 ± 4.72	5.21 ± 4.79	5.39 ± 4.91	0.76
	0–28 d	25 (1.62%)	57 (4.48%)	15 (5.62%)	5 (2.5%)	
	29 d–12 mo	382 (24.81%)	258 (20.27%)	59 (22.10%)	36 (18%)	
	1–7-y	717 (46.56%)	561 (44.07%)	111 (41.57%)	84 (42%)	
	8–18 y	416 (27.01%)	397 (31.19%)	82 (30.71%)	75 (37.5%)	
**Sex**	Males	889 (57.72%)	732 (57.50%)	157 (58.80%)	117 (58.5%)	0.80
	Females	651(42.27%)	541 (42.50%)	110 (41.20%)	83 (41.5%)	
**Weight (kg)**		23.74 ± 19.09	23.71 ± 18.86	23.92 ± 20.23	29.41 ± 21.77	0.87
**Height (cm)**		105.02 ± 36.02	105.27 ± 35.88	103.89 ± 36.81	113.38 ± 37.02	0.57

Data are presented as number (%) and median ± standard deviation.

D, days; mo, months

The incidence of hypoxemia was higher among younger patients. Twenty-five of 58 (43.10%) neonates, 382 of 1,443 (26.47%) infants, 717 of 6,888 (10.41%) patients aged 1–7 years, and 416 of 4,741 (8.77%) children aged 8–16 years experienced hypoxemic events in the intraoperative period.

Of the 2,367 episodes of intraoperative hypoxemia, 1,166 (49.26%) occurred during emergence from anesthesia, 772 (32.62%) during anesthesia maintenance, and 429 (18.12%) during the induction period. Further, 232 patients (15.06%) experienced hypoxemic events more than twice during surgery. The median duration of hypoxemia per episode was 55 s (95% CI 43 to 67 s).

In total, 200 patients’ records out of 289 hypoxemia episodes were used for holdout independent validation dataset. Five of 12 (41.67%) neonates, 36 of 159 (22.64%) infants, 84 of 663 (12.67%) patients aged 1–7 years, and 75 of 672 (11.16%) children aged 8–16 years had a hypoxemic event during the intraoperative period. During each of the three time intervals, which are induction, maintenance, and emergence, 48 (16.61%), 44 (15.22%), and 197 (68.17%) episodes, respectively were identified. Patients with intraoperative hypoxemia events in the holdout dataset had a median duration of a hypoxemic event of 50 s (95% CI 45 to 55 s).

### Model performance in predicting hypoxemia

After the hyperparameter search of each machine learning and deep learning algorithm, the optimal hyperparameters for each model were as follows: GBM: {number of trees: [2000], max depth: [5], subsampling rate: [0.5]}, LSTM: {number of hidden layers: [1], number of hidden nodes: [64], number of dense nodes: [16], dropout rate: [0.5]}, Transformer: {number of filters: [64], number of heads: [3], embedded dimension: [32], number of convolutional layer: [1], number of transformer layers: [3], dropout rate: [0.2]}. The models were evaluated using the best performing settings.

**Fig 2:**
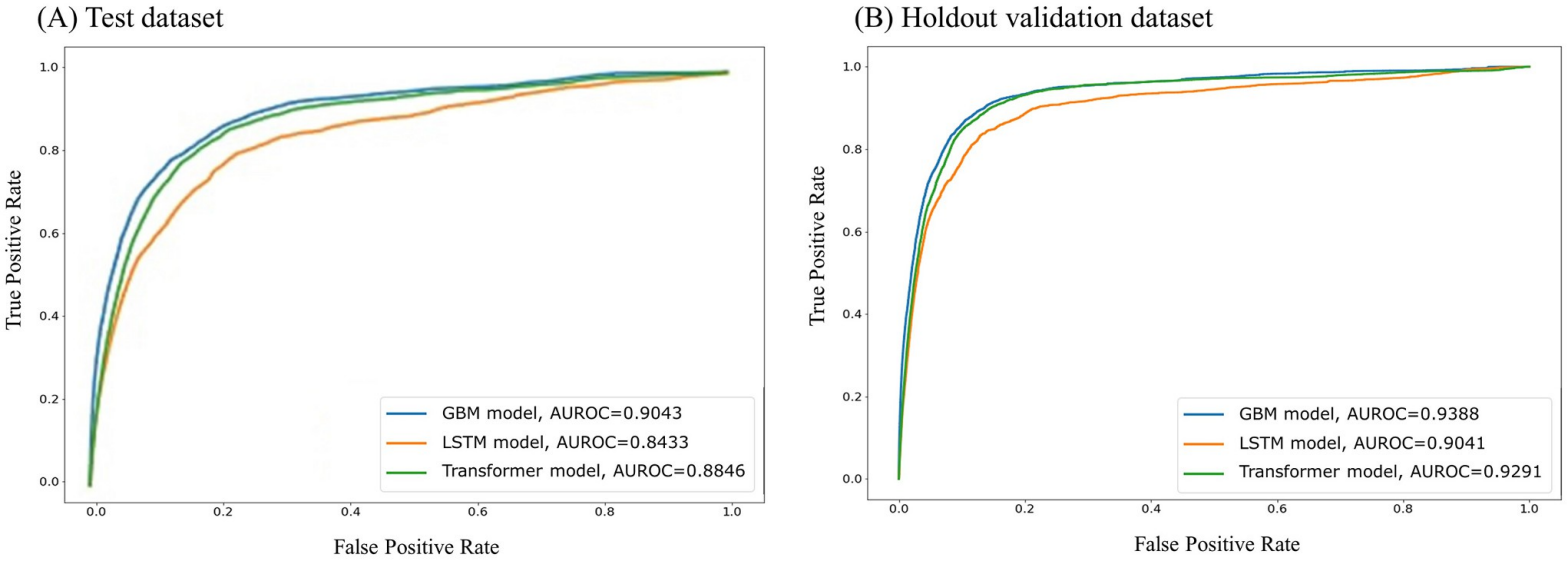
Comparison of area under the receiver operating characteristic curves for the machine learning models for predicting intraoperative hypoxemia in pediatric patients in (A) test dataset (B) holdout validation dataset. GBM, Gradient-boosting model; LSTM, Long short-term memory; AUROC, Area under the receiver operating characteristic curve.

ROC curves for the ability of each model to predict hypoxemia 60 s prior to the measurement point are presented in Fig 2(A). Table 2 describes the predictive performance of the three modeling approaches. Patient weight, SpO_2_, EtCO_2_, FiO_2_, TV, and PIP were used as input variables for performance implementation (S1 Fig). All three models demonstrated good predictive ability (AUROC ≥0.7). Among the developed models, the GBM model had the highest AUROC value of 0.934 (95% CI 0.902 to 0.906), which was significantly higher than that of the LSTM model (0.851, 95% CI 0.840 to 0.846, *P*<0.001) and the transformer model (0.875, 95% CI 0.882 to 0.887, *P*<0.001).

**Table 2:** Ability of models in predicting intraoperative hypoxemia in pediatric patients.

Models	Test dataset (model development)			Holdout validation dataset		
	AUROC [CI]	AUPRC	P-value	AUROC [CI]	AUPRC	P-value
**GBM**	0.904 [0.902–0.906]	0.225		0.939 [0.936–0.941]	0.235	
**LSTM**	0.843 [0.840–0.846]	0.106	<0.05	0.904 [0.900–0.907]	0.124	<0.05
**Transformer**	0.897 [0.882–0.887]	0.121	<0.05	0.929 [0.926–0.932]	0.145	<0.05

Abbreviations: AUROC, area under the receiver operating curve; AUPRC, area under the precision-recall curve; CI, confidence interval; GBM, gradient-boosting model; LSTM, long short-term memo

**Fig 3:**
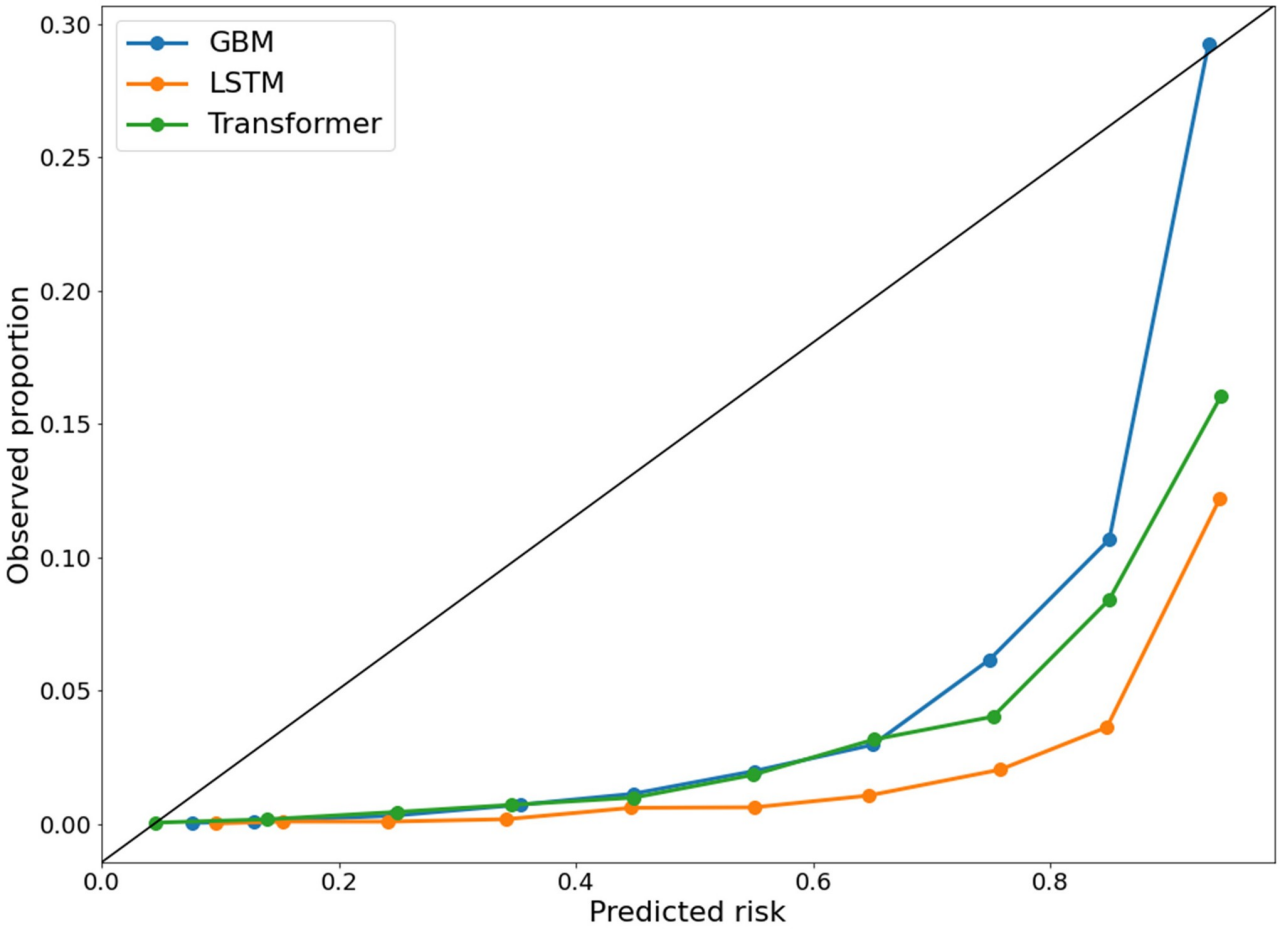
Calibration plot of the prediction in the holdout validation cohort. GBM, Gradient-boosting model; LSTM, Long short-term memory.

The best performing models were also evaluated on an independent holdout validation cohort. Hypoxemic events were identified in the records of 200 (13.25%) patients with 289 episodes out of 1,510 patients in the holdout validation cohort. Similar to our model development, GBM also demonstrated best performance with an AUROC of 0.939 (95% CI 0.936 to 0.941, sensitivity of 0.855, and specificity of 0.807), which was better than LSTM (AUROC of 0.904, 95% CI, 0.900–0.907, sensitivity of 0.798, specificity of 0.775, *P*<0.001) and transformer (AUROC of 0.929, 95% CI 0.926 to 0.932, sensitivity of 0.849, specificity of 0.784, *P*<0.001). In the calibration plot analysis in the holdout validation cohort (Fig 3), the GBM model showed the best calibration performance among the three models; however, the models demonstrated overestimated risks. Overall, as indicated in Fig 2(B), all the three models were highly predictive of intraoperative hypoxemia in the validation cohort (AUROC values ≥0.7), with the AUROCs ranging from 0.904 to 0.939. Moreover, in both the assessments, GBM model showed better AUPRC score than the other methods and confirmed that they worked well in imbalanced hypoxemia predictions as shown in Table 2.

## Discussion

This study aimed to develop a machine learning model that predicts hypoxia 1 min prior during general anesthesia in pediatric patients and verify the performance of our model using a pediatric vital signs registry dataset. Our results demonstrated that hypoxemia in pediatric patients undergoing general anesthesia can be predicted with a deep learning model using pulse oximetry data and ventilator parameters. All the three trained machine learning models exhibited high prediction performance, while GBM showed the best performance.

Respiratory complications derived from intraoperative desaturation are the most frequent adverse events in pediatric anesthesia [2]. Highly variable incidences of intraoperative hypoxemia in pediatric patients have been reported (1–22%) [2, 25, 26]. A previous study estimated the incidence of intraoperative hypoxemia (SpO_2_ ≤95%) in pediatric surgical patients aged 0–16 years to be 10%, which is comparable to our study [5]. A recent prospective observational study demonstrated the crude overall incidence for desaturation (SpO_2_ ≤95%) in pediatric surgical patients to be 23.8% [3]. Our study results are consistent with previous reports that the probability of desaturation episodes is increased in younger age groups and during the emergence period [3]. Compared to other studies, a relatively low proportion of hypoxemic incidents occurred during induction [3]. This difference is derived from the characteristics of the study setting, i.e., a general tertiary teaching hospital, intravenous induction in most cases. First, in our institution, most children undergo intravenous induction, except for patients with failed intravenous cannulation in the ward or day center. According to a previous randomized controlled trial study and meta-analysis, children receiving intravenous induction agents were significantly less likely to experience perioperative respiratory adverse events, including desaturation (SpO_2_ < 95%), compared to those who received inhalational anesthesia induction [27, 28]. Second, our center commences induction in the setting of FiO_2_ 1.0; thus, the low proportion of induction-related hypoxemia compared to other institutions cannot be ruled out. Moreover, as intraoperative hypoxemia is known to occur more often in the induction period, a greater number of pediatric anesthesiologists with >2 years’ experience in pediatric anesthesia are involved in that period to ensure more meticulous management in our institute [8, 29]. On the other hand, during the emergence period, a single anesthesiologist is usually in charge owing to the busy schedule in the hospital; thus, the response to hypoxemia is considered relatively inadequate. The difference presumably occurs in terms of preventing hypoxemia in induction period since greater manpower is involved in solving the hypoxemia at the time of emergence. Lastly, unlike other studies reporting intraoperative hypoxemia, the duration of hypoxemia was not considered in this study; our study defined hypoxemia as peripheral oxygen saturation below 95% regardless of its duration [2, 6, 29]. Thus, we carefully suggest that the high incidence of hypoxemia during the emergence period could be underreported for a very brief hypoxemia episodes in previous studies [2, 6, 29].

Perioperative respiratory adverse events are common in pediatric anesthesia, which could result in hypoxemia [30]. However, no standardized definition of perioperative respiratory adverse events exists in the literature; nevertheless, perioperative hypoxemia is often defined as the drop of the value of SpO_2_ under 95% [29]. Therefore, we set 95% as a threshold point for the risk of hypoxemia in children considering the provision of a relatively high FiO_2_ during general anesthesia, the faster progression of hypoxemia relative to that in adults, and our purpose of developing a predictive model for rapid recognition of the risk of further desaturation and more applicable intervention in clinical setting.

In our study, we adopted EtCO_2,_ FiO_2,_ TV, and PIP as input variables for machine learning performance improvement. In many cases, hypoxemic events in children in general anesthesia setting tend to be associated with extrinsic events, such as challenging intubation, circuit disconnection, accidental one-lung ventilation, large amount of secretions or tube kinking, inadvertent extubation, and laryngospasm. These situations generally cause a change in the ventilator parameters, which are mostly elevated airway pressure, reduced TV, and abrupt change of EtCO_2_. Moreover, the higher probability of occurrence and faster onset of a desaturation events were observed at a lower FiO_2_ compared to when the FiO2 is 1.0 [31]. This model using interpretable input variables provides the detection of subtle changes that clinicians could not be easily aware of and comprehensive integration of high-fidelity operating room data. Although relatively low AUPRC of our models would have resulted in multiple false positive predictions, reducing the risk of exposure to hypoxemia, thereby outweighing the alarm fatigues experienced by anesthesiologists. Ultimately, predictive models using machine learning techniques could be developed to build real-time guided additional instructions for intraoperative hypoxemia management.

We developed three applicable machine learning algorithms with good performance regardless of the patient’s age and periods of anesthesia (induction, maintenance, and emergence). The extracted features of vital sign and ventilator parameters from operating room data have a variety of complex nonlinear interactions, which is necessary to build a model with significant flexibility. GBM is an ensemble forward learning model that is a nonparametric model comprising an ensemble of weak prediction models for the development of a final model. It has the ability to incorporate these large amounts of disparate data into a unified algorithm, which is similar to the principle and decision making process of anesthesiologists do in clinical practices [32]. Moreover, large datasets can boost the prediction performance of GBM by imploring an additional classifier [33]. LSTM is a type of recurrent neural network (RNN) models which deal with time series analysis that processes inputs and outputs as data units of related sequences [34]. The model was selected and developed with the goal of real-time prediction of hypoxemia through time series analysis using input signals of vital signs and ventilator parameters in a clinical setting. Transformer processes entire sequence of data and uses self-attention mechanisms to learn dependencies in the sequence. It is known to have potential to learn complex dependencies of various length from time series data, which showed moderate performance of predicting intraoperative hypoxemia [35].

Recently, machine learning techniques analyzing biosignal waveforms have been increasingly developed for the clinical prediction of medical conditions [13, 16, 36]. A previous group reported the development of a machine learning-based intraoperative hypoxemia prediction system for adult patients during general anesthesia [14]. However, this study was limited since the authors used minute-by-minute intervals for the model training [14]. In our study, biosignal waveforms were used for the dataset from Vital Recorder, thus providing real-time graphical capnography, and the ventilator parameters reflect the clinical changes per second. This high-fidelity and large second-by-second datasets could help improve the accuracy of hypoxemia prediction models. This analysis enables more rapid detection of upcoming hypoxemia, which is particularly important in children. Considering that the median duration of intraoperative hypoxemia was 55 s, i.e., <1 min, it is believed that a prediction model using an input dataset with as short as possible time interval would have greater clinical significance.

However, the present study had several limitations. First, although we screened over 14,000 surgical cases, intraoperative hypoxemia is a relatively rare event, which limited the number of cases included in the final analysis. In addition, we only included pediatric patients who underwent surgery under general anesthesia at a single center and thereby could not externally validate the data. However, our institution accounts for the largest number of pediatric surgeries in the country each year, which makes it possible to obtain a large number of data for developing applicable algorithms. Moreover, all the three machine learning models developed in this study showed high performance in the holdout validation cohort based on the AUROC. Furthermore, the calibration plot performance from all machine learning models were poor with an overestimated risk, which might results in overtreatment. The main cause of poor calibration is the lack of data, which could be solved by using more datasets [24]. Further validation in other centers is warranted to ensure the consistency and robustness of our findings. Moreover, we excluded various types of high-risk surgeries associated with a higher incidence of hypoxemia, such as cardiothoracic surgery. Furthermore, risk stratification for children who had significant comorbidities and were prone to encounter desaturation events was not considered. Several clinical diagnoses associated with intraoperative hypoxemia, such as accidental one-lung ventilation, tracheal balloon leak, endotracheal tube obstruction, and patient factors including pulmonary disease, are not directly observable and are strongly subjective; thus, they are difficult to transit into datasets that can be directly utilized [14]. We did not identify and stratify factors showing a predictive role for intraoperative hypoxemia during general anesthesia in children. Finally, the potential benefits of prediction using machine learning techniques to anesthesiologists in the operating room were not evaluated.

## Conclusion

The machine learning algorithm using demographic, vital sign, and other ventilatory data can predict hypoxemia in children under general anesthesia. If machine learning-based prediction models are successfully applied in clinical practice, patient outcomes would be improved by acquiring more time for patient management and implementation of earlier management. Future studies focusing on whether the use of machine learning-based prediction in the clinical workflow achieves superior performance compared to practicing anesthesiologists are warranted.

## Supporting information

S1 Fig Simplified plot of the real-time prediction of intraoperative hypoxemia. (PDF)

## Abbreviations

AUC: area under the curve
AUPRC: area under the precision-recall curve
AUROC: area under the receiver operating curve
CI: confidence interval
EtCO_2_: end tidal CO_2_
FiO_2_: fraction of inspired oxygen
GBM: gradient-boosting model
LSTM: long short-term memory
PIP: peak inspiratory pressure
SpO_2_: oxygen saturation
TV: tidal volume
