# Supplementary material for: Machine learning-based prediction of intraoperative hypoxemia for pediatric patients
Source: PLoS One. 2023 Mar 1;18(3):e0282303. doi: 10.1371/journal.pone.0282303 (PMC9977036; doi:10.1371/journal.pone.0282303)
Supplement: S1 Fig — (PDF) [file pone.0282303.s001.pdf]

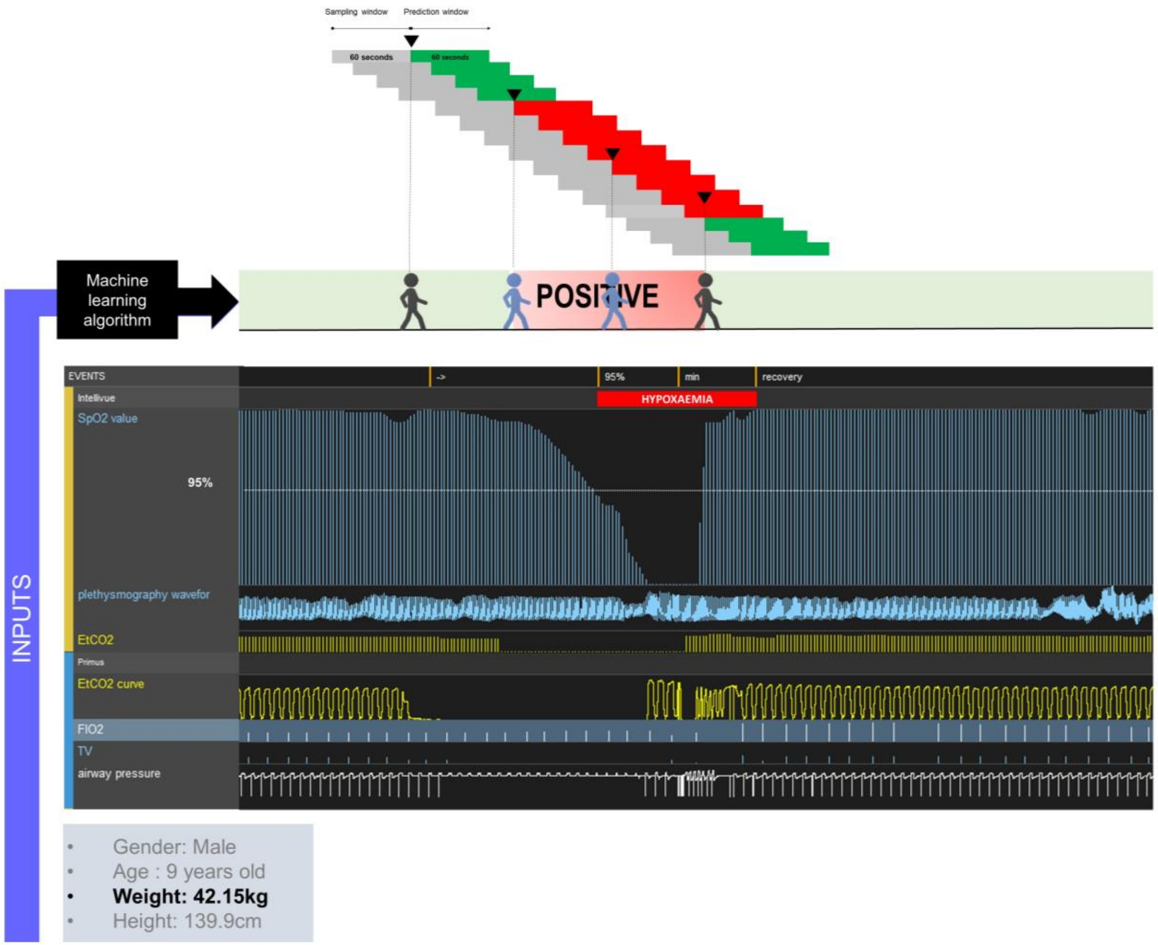

Supplementary Figure 1. Simplified plot of the real-time prediction of intraoperative hypoxemia. Predictions are performed in a moving window which incrementally advances in 2-second intervals. Owing to the intrinsic limitations of GBM of being unable to consider time-series data, only the first event of each case was used for training and testing. $EtCO_2$, End tidal CO_2; FiO_2, Fraction of inspired oxygen; GBM, Gradient-boosting model
